# Simultaneous Raman and Infrared Spectroscopy of Stable Isotope Labelled *Escherichia coli*

**DOI:** 10.3390/s22103928

**Published:** 2022-05-22

**Authors:** Cassio Lima, Howbeer Muhamadali, Royston Goodacre

**Affiliations:** Centre for Metabolomics Research, Department of Biochemistry and Systems Biology, Institute of Systems, Molecular and Integrative Biology, University of Liverpool, Liverpool L69 7ZB, UK; cassio.lima@liverpool.ac.uk (C.L.); howbeer.muhamad-ali@liverpool.ac.uk (H.M.)

**Keywords:** Raman, O-PTIR, bacteria, stable isotope labeling, spectroscopy

## Abstract

We report the use of a novel technology based on optical photothermal infrared (O-PTIR) spectroscopy for obtaining simultaneous infrared and Raman spectra from the same location of the sample allowing us to study bacterial metabolism by monitoring the incorporation of ^13^C- and ^15^N-labeled compounds. Infrared data obtained from bulk populations and single cells via O-PTIR spectroscopy were compared to conventional Fourier transform infrared (FTIR) spectroscopy in order to evaluate the reproducibility of the results achieved by all three approaches. Raman spectra acquired were concomitant with infrared data from bulk populations as well as infrared spectra collected from single cells, and were subjected to principal component analysis in order to evaluate any specific separation resulting from the isotopic incorporation. Similar clustering patterns were observed in infrared data acquired from single cells via O-PTIR spectroscopy as well as from bulk populations via FTIR and O-PTIR spectroscopies, indicating full incorporation of heavy isotopes by the bacteria. Satisfactory discrimination between unlabeled (*viz*. ^12^C^14^N), ^13^C^14^N- and ^13^C^15^N-labeled bacteria was also obtained using Raman spectra from bulk populations. In this report, we also discuss the limitations of O-PTIR technology to acquire Raman data from single bacterial cells (with typical dimensions of 1 × 2 µm) as well as spectral artifacts induced by thermal damage when analyzing very small amounts of biomass (a bacterium tipically weighs ~ 1 pg).

## 1. Introduction

Vibrational spectroscopy, including Raman and infrared modalities, provide molecular information in a rapid and high-throughput way by probing spectral signatures of (bio)chemical species from a wide range of samples [1]. Both techniques have been extensively applied over the years to characterize specimens in chemistry, material sciences, and biological sciences in a non-invasive, non-destructive, and label-free manner [1]. Due to their unique “fingerprinting” capabilities, Raman and infrared spectroscopies have been intensively used as whole-organism fingerprinting tools in microbial metabolomics studies to investigate the wide spectrum of phenotypic traits expressed by microbes, providing deeper insights into microbial cell physiology as well as a better understanding on the impacts of different environmental conditions to microbial cell functions and, ultimately, an assessment of the overall interactions between the microbial cells’ host-environment [2,3,4,5].

Although Raman and infrared spectroscopy provide molecular information based on the interaction of electromagnetic radiation with matter, there are pros and cons regarding their use for analyzing biological samples, as both techniques operate differently [6,7]. In Raman spectroscopy, photons from a laser source interact with chemical species from samples and are scattered with different energies [8]. Most modern Raman systems operate using lasers ranging from UV into the near-infrared as the excitation source, which provide photons with an energy that may coincide with molecular absorption bands from metabolites (antibacterials, antifungals, vitamins, enzymes, and pigments) produced by many microorganisms such as yeasts and bacteria [9]. Thus, one of the main challenges regarding the use of Raman spectroscopy to study microbial samples is the interfering baseline signal from laser-induced fluorescence competing with the Raman signal [8]. By contrast, the frequency coincidence (resonance) of incident photons to the energy required to induce electronic transitions may lead to enhancement of Raman scattering (resonance Raman), which is particularly advantageous when analyzing metabolites in low concentrations where signatures are usually not observed in the spectrum achieved by the non-resonant Raman process due to weak Raman scattering (only 1 in 10^8^ photons are inelastically scattered) [8]. Infrared spectroscopy is based on the absorption of infrared radiation by molecular vibrations, so unlike Raman, fluorescence is not an issue as photons in the IR part of the electromagnetic spectrum do not have enough energy to induce electronic transitions. However, the main drawback of infrared spectroscopy is the interference from water as infrared radiation is strongly absorbed by water, and hence sample drying steps are necessary to avoid interference from water signatures, which generally makes infrared spectroscopy unsuitable for studying living systems [6,10].

Raman and infrared spectroscopies have been extensively used separately as phenotyping tools in a wide range of microbial metabolomics investigations including studying the effects of antibiotics in bacteria [11,12], classifying and identifying microbial species and strains [2,3,13], effects of environmental factors on microbial growth [14,15], and microbial-derived products such as beverages [16,17], biofuels [18,19] and other products for human and animal health. Both methods are often used together in the same study in a complementary way as each technique may be advantageous when studying certain types of samples or biological processes as a result of their different basic working principles [3,14,20,21,22]. In such cases, two independent instruments are required as acquiring Raman and infrared spectra simultaneously using one single system has been a challenge due to technical difficulties regarding sharing optics and light sources in a conventional Raman and infrared spectrometers [23]. More recently, a novel spectroscopic approach, so-called Optical Photothermal infrared (O-PTIR) spectroscopy, has been developed aiming to collect an infrared spectrum at sub-micron spatial resolution without relying on evanescent light unlike other infrared methods based on near-field optics. In this pump–probe scheme, a tunable mid-IR quantum cascade laser (QCL) excites the sample (pump) while a 532 nm continuous wave (CW) laser interrogates the sample (probe). The infrared radiation delivered by the QCL beam induces thermal expansion due to local temperature rise, whereas the CW beam probes the induced photothermal effect. Simultaneously, the probe beam also acts as the excitation wavelength to acquire Raman spectra from the same sample location at the same resolution [24,25,26]. In addition to providing Raman and infrared signatures simultaneously, O-PTIR spectroscopy provides an infrared fingerprint signal with a submicron resolution, which opens up opportunities to perform microbial metabolomics investigations at the single-cell level [25]. Although FTIR spectroscopy has been successfully applied for phenotyping microbial samples, most studies have been focused on bulk measurements due to the poor resolution achieved by a state-of-the-art FTIR spectrometer (3–30 μm) [12,25]. Thus, considering that bulk measurements can average out important information about microbial communities, O-PTIR spectroscopy emerges as a new phenotyping tool allowing to study cell metabolism, including cell-to-cell variance. In light of this, here we report the use of O-PTIR technology as a tool to study bacterial metabolism by monitoring the incorporation of ^13^C- and ^15^N-labeled compounds through Raman and infrared spectral signatures collected simultaneously from bacterial bulk populations and single cells. Incubation of bacteria with stable isotope-labeled substrates combined with Raman and infrared spectroscopy has been used extensively over the years to provide insights into the function of bacteria. Although the effects of isotopic labeling on Raman spectra acquired from bulk populations and single cells are well known, there are no studies comparing the effects induced by the incorporation of heavy atoms in the infrared signatures acquired from single bacterial cells and bulk populations.

## 2. Materials and Methods

### 2.1. Growth Conditions and Sample Preparation

*Escherichia coli* K-12 MG1655 cells were cultivated on LB agar at 37 °C for 24 h. Bacterial inoculum was prepared by transferring a single colony from the LB agar plates into sterile 24-well plates (Greiner Bio-one, Stonehouse, UK) containing minimal medium supplemented with unlabeled glucose (^12^C) or isotope-labeled glucose (^13^C) (99 atom % homogeneously labeled; from Sigma-Aldrich, Gillingham, UK) as the source for carbon (5 g/L) as well as with unlabeled ammonium chloride (^14^N) or isotope-labeled ammonium chloride (^15^N) (99 atom % homogeneously labeled; from Sigma-Aldrich, Gillingham, UK) as a source for nitrogen (1 g/L), therefore resulting in three experimental groups (^12^C^14^N, ^13^C^14^N, and ^13^C^15^N). After the incubation period, samples were prepared following standard protocols for spectroscopy previously published by our group [25].

### 2.2. FTIR Spectroscopy

FTIR spectral data in the mid-IR range (4000–600 cm^−1^) were acquired from bulk populations using a Bruker Invenio FTIR spectrometer equipped with high-throughput measurements (HTS-XT) (Bruker Ltd., Coventry, UK) accessory. In total, 20 µL aliquots from each sample were transferred to a silicon substrate (Bruker Ltd., Coventry, UK) and dried for 30 min in an oven at 55 °C [3]. Each spectrum results from 64 accumulations with 4 cm^−1^ of spectral resolution. In total, 20 spectra were obtained for each experimental group from 5 analytical replicates per group (4 spectra per analytical replicate).

### 2.3. O-PTIR and Raman Spectroscopy

O-PTIR measurements were collected from bulk samples and individual cells. For bulk samples, 2 µL aliquots from each sample were spotted onto a calcium fluoride (CaF_2_) substrate. For individual cells, samples were diluted in deionized water (1:1000) and 2 µL aliquots were transferred to CaF_2_ slides. Both bulk and single-cell samples were dried in the oven prior to analysis as described above. Single-point O-PTIR spectra were collected in reflection mode over the spectral region of 950−1800 cm^−1^ using a mIRage microscope (Photothermal Spectroscopy Corp., Santa Barbara, CA, USA). Spectra were recorded with a spectral resolution of 2 cm^−1^ and 10 accumulations per spectrum. The pump and probe power used for acquiring data from bulk populations were 1.75 and 48 mW, while 6.25 and 48 mW were used for single cells, respectively. Raman scattering was detected after photons passed through the Cassegrain 40× objective (0.78 NA) into a Horiba iHR320 spectrometer equipped with a 600 groove/mm grating. Raman spectra were collected from bulk populations over the range of 3500−500 cm^−1^ using 48 mW as probe power, an integration time of 10 s, 10 accumulations and 4 cm^−1^ spectral resolution.

### 2.4. Data Analysis

All pre-processing steps and spectral analysis were carried out in MATLAB 2011a (MathWorks Inc., Natwick, MA, USA). Infrared and Raman data underwent baseline correction using an algorithm based on asymmetric least squares, then smoothed via Savitzky−Golay filter (2nd order polynomial; 11-point window) and vector normalized. Next, spectral data were subjected to principal component analysis (PCA). 

## 3. Results and Discussion

**Figure 1:**
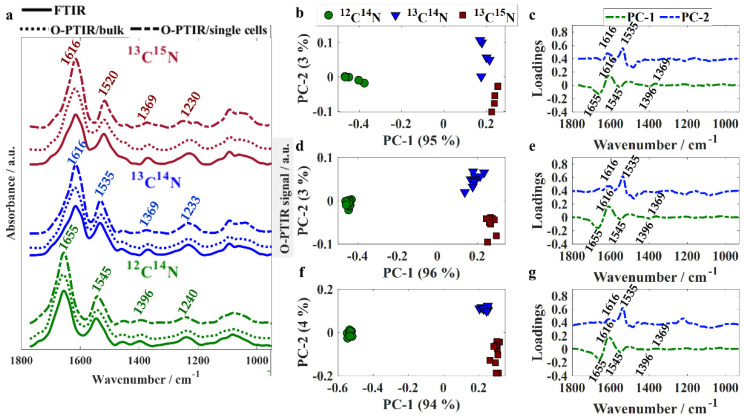
(**a**) Fingerprint region of averaged spectra collected via FITR, O-PTIR/bulk, and O-PTIR/single cells acquired from *E. coli* cells incubated with ^12^C^14^N (green line), ^13^C^14^N (blue line), and ^13^C^15^N (red line). Plots are offset for clarity. PCA scores and loadings plots obtained using FTIR (**b**,**c**), O-PTIR/bulk (**d**,**e**), and O-PTIR/single cells (**f**,**g**) as input data; values in parentheses are the percentage of total explained variance (TEV).

Infrared spectral data recorded from unlabeled and isotopically labeled bacteria via FTIR and O-PTIR spectroscopies were compared in order to evaluate the ability of the two technologies to probe the expression of phenotypic traits displayed by bacterial bulk populations and individual single cells. Infrared signatures acquired by FTIR spectroscopy were recorded from 400–4000 cm^−1^; however, only the fingerprint region was assessed as the bands peaking at the high wavenumber region (2800–3200 cm^−1^) are not accessible by the O-PTIR spectrometer employed in the present study, as the QCLs used for excitation cover only the fingerprint region (950–1800 cm^−1^). To the best of our knowledge, there is no tunable laser that covers the whole mid-IR spectral range; however, a tunable OPO-pulsed infrared laser can be added to the system in order to collect infrared signatures in the high wavenumber region [26]. Figure 1a shows the fingerprint region (950–1800 cm^−1^) from averaged infrared spectra acquired from bulk populations via FTIR and O-PTIR spectroscopies as well as from individual single cells through O-PTIR spectroscopy.

Although O-PTIR spectroscopy is performed on a localized spot of a single bacterium, whereas measurements from bulk populations result from the average signal collected from several bacteria illuminated by the beam, significant similarities and high data reproducibility were observed in spectral signatures acquired from single cells via O-PTIR spectroscopy and bulk populations via both O-PTIR and FTIR (Figure 1a) spectroscopies. In previous studies published by our group, we showed that spectral signatures acquired from bulk populations via FTIR spectroscopy and single cells via O-PTIR spectroscopy exhibited shifts in the peak position from bands arising from proteins as a result of the incorporation of heavy isotopes [22,25]. The band peaking at 1655 cm^−1^ in unlabeled bacteria (amide I vibration [27]) arises mainly from the contribution of C=O stretching from peptide bonds and displays a shift of 39 cm^−1^ towards lower wavenumbers (1616 cm^−1^) in isotopically labeled cells due to the heavier ^13^C isotope replacing the ^12^C in the C=O functional group. The band peaking at 1545 cm^−1^ in unlabeled cells is attributed to amide II vibrational mode, which results from the combination of vibrations from multiple chemical species with the main one being the out-of-phase N−H in-plane bend coupled to C−N stretching from peptide groups [27]. In ^13^C^14^N-labeled cells, the amide II band is seen peaking at 1535 cm^−1^ as a result of the uptake of ^13^C atoms from ^13^C-labeled glucose used as the carbon source. In ^13^C^15^N-labeled samples, the shift observed for this band was even greater due to the incorporation of heavier carbon (^13^C) and nitrogen (^15^N) atoms from both ^13^C-glucose and ^15^N-amonnium chloride. The band observed in 1396 cm^−1^ in unlabeled bacteria is attributed to amino acid chains and carboxylic acids [13,28,29] and displayed a shift of 27 cm^−1^ towards the lower wavenumber region in ^13^C-labeled bacteria. Amide III vibration (1240 cm^−1^ in unlabeled bacteria) [27] is also affected by the uptake of heavy isotopes as it results from the combination of C−N and N−H vibrational modes (^13^C^14^N: 1233 cm^−1^; ^13^C^15^N: 1230 cm^−1^). Infrared spectra collected through all three different approaches (FTIR/bulk, O-PTIR/bulk, and O-PTIR/single cells) were used as input to PCA in order to assess the clustering patterns. Similar clustering patterns were observed in the scores plots obtained using data acquired through all three approaches as the inputs (Figure 1b,d,f). Scores from unlabeled bacteria were clustered on the negative side of PC-1 axis, whereas scores obtained from spectra from labeled bacteria (^13^C^14^N and ^13^C^15^N) grouped along the positive side; this illustrates the dominance of carbon-based vibrations in bacterial cells as this shift was in the first PC which by definition is extracted to explain the most natural variance in the spectra (94–96% total explained variance (TEV)). PC-1 illustrated negative loadings at 1655, 1545, and 1396 cm^−1^ and positive loadings for 1616 and 1369 cm^−1^ (Figure 1c,e,g). On the PC-2 axis, scores from ^13^C^14^N-labeled bacteria clustered on the positive values, while scores from ^13^C^15^N-labeled cells can be observed along the negative axis. The loadings indicated that discrimination displayed in PC-2 is due to nitrogen based functional groups from amide II vibrations (1535 cm^−1^ in ^13^C^14^N-labeled bacteria).

**Figure 2:**
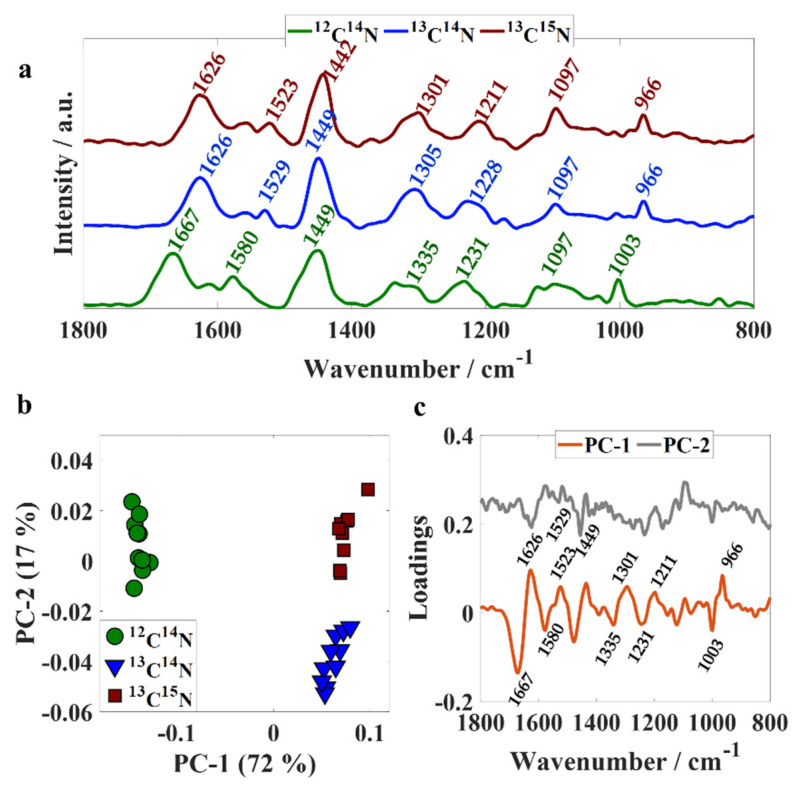
(**a**) Fingerprint region of averaged Raman spectra collected from *E. coli* bulk populations simultaneously with infrared data using O-PTIR system; ^12^C^14^N (green line), ^13^C^14^N (blue line), and ^13^C^15^N (red line). Plots are offset for clarity. (**b**) PC scores plot; values in parentheses are the percentage total explained variance (TEV). (**c**) PC loading plot.

Although Raman spectroscopy using 532 nm as the excitation wavelength has been successfully applied to collect Raman signatures from a single bacterium in previous studies [22], we did not succeed in collecting Raman spectrum from single cells concomitant with infrared data using O-PTIR technology; therefore, the present study focused on analyzing Raman signatures acquired from bulk samples and not individual cells. Figure 2a illustrates Raman spectral data from unlabeled and isotopically labeled bulk populations recorded simultaneously with infrared data via the O-PTIR system. The band peaking at 1667 cm^−1^ (amide I) in unlabeled bacteria arises from C=O stretching from peptide bonds [4] and shifted 41 cm^−1^ towards lower wavenumbers (1626 cm^−1^) upon ^13^C and ^15^N incorporation in ^13^C^14^N- and ^13^C^15^N-labeled cells. The fact that there is no shift on the position of this peak in both infrared and Raman signatures from ^13^C^14^N- and ^13^C^15^N-labeled samples suggests that this vibrational mode is dominated mainly by C=O stretching and is minimally/not affected by molecular bonds with nitrogen as it has been suggested by previous publications [27]. The band peaking at 1580 cm^−1^ corresponds to amide II vibration from proteins in unlabeled cells [4], which shifted to 1529 cm^−1^ in ^13^C^14^N-labeled bacteria and 1523 cm^−1^ in ^13^C^15^N-labeled samples, indicating that this vibrational mode is affected by the uptake of both ^13^C and ^15^N heavy isotopes due to the combined effects of the incorporation of heavier atoms on the C−N and N−H vibrational frequencies. Similar findings were obtained for vibrational modes associated with the amide III band from proteins, which illustrates a band peaking at 1231 cm^−1^ in unlabeled bacteria, 1228 and 1211 cm^−1^ in ^13^C^14^N- and ^13^C^15^N-labeled samples [4]. The higher shift obtained from ^13^C^15^N-labeled cells (20 cm^−1^) indicates that this vibrational mode is mainly affected by molecular bonds containing nitrogen atoms. The band peaking at 1335 cm^−1^ in unlabeled cells arises from nucleic acids and displayed a shift of 30 cm^−1^ towards lower wavenumbers in ^13^C^14^N-labeled bacteria, and 34 cm^−1^ in ^13^C^15^N-labeled cells [22]. The vibration observed at 1003 cm^−1^ in unlabeled bacteria is attributed to the ring breathing of phenylalanine [4,22], which shifted to 966 cm^−1^ in ^13^C^14^N-and ^13^C^15^N-labeled samples. Figure 2b displays the scores plot obtained by subjecting Raman spectral data to PCA, which illustrates clear discrimination between unlabeled, ^13^C^14^N-and ^13^C^15^N-labeled bacteria. Scores from unlabeled cells were grouped along negative axis of PC-1 and, consequently, PC-1 illustrated negative loadings at 1667, 1580, 1335, 1231, and 1003 cm^−1^ and positive loadings for 1626, 1523, 1301, 1211, and 966 cm^−1^. On the PC-2 axis, scores from ^13^C^14^N-labeled bacteria can be seen on the negative side, while most scores were associated with spectra acquired from ^13^C^15^N-labeled bacteria clustered on the positive axis. Loading assessment shows that the clustering pattern displayed on PC-2 is mainly due to differences on peak intensity as we observed negative loadings at 1626, 1529, and 1449 cm^−1^ from ^13^C^14^N-labeled bacteria.

**Figure 3:**
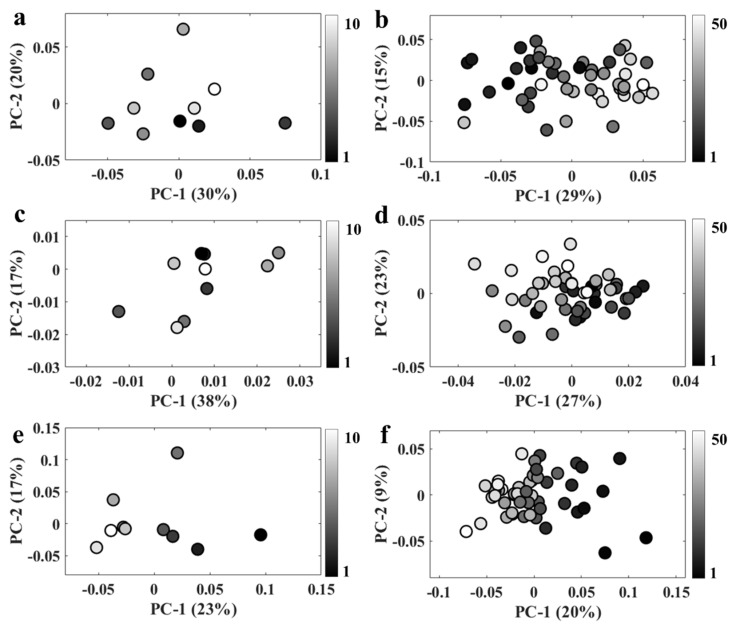
PC scores plot obtained from spectra acquired from the same spot. (**a**,**b**) 10 and 50 infrared spectra from a single bacterium; (**c**,**d**) 10 and 50 infrared spectra obtained from bulk; (**e**,**f**) 10 and 50 Raman spectra collected from bulk populations. The grey-scale color of the symbols relates to the number of repeat spectral acquisitions from the same spots.

Acquiring Raman and infrared spectra simultaneously with an O-PTIR system requires illuminating the sample with two lasers with relatively high energies, which may induce changes in spectral signatures due to thermal damage or even total cell degradation. In order to test whether repeated analysis may affect the spectral quality, 50 Raman and infrared spectra were acquired from the same spot from bulk populations and a single bacterium in order to evaluate the changes induced in the spectral signatures due to the increase in local temperature. Figure 3 displays PCA scores plots obtained by applying PCA on O-PTIR data collected from a single bacterium (Figure 3a,b) as well as O-PTIR (Figure 3c,d) and Raman (Figure 3e,f) spectra acquired from bulk populations. Figure 3a,c,e shows the PCA results applied to the first 10 spectra, while Figure 3b,d,f illustrates the results obtained using all 50 spectra as the input data for PCA. Scores plots displayed in Figure 3b,d exhibited a bit of a trend on the PC-1 axis, as most spectra acquired at the beginning of the time-series scan were clustered on the negative side of PC-1, while the data acquired at the end of the scan grouped along positive side of PC-1 axis. These findings indicate that the first 10 infrared spectra acquired from bulk populations and single bacterium are not affected by the pump and probe laser energies (especially as no trends are seen in Figure 3a,c from the first 10 O-PTIR analyses); however, spectral changes might be expected after acquiring 50 infrared spectra from both bulk and individual cells. For Raman data, spectral changes are very clearly seen for the first 10 (Figure 3e) as well as all 50 analyses (Figure 3f), and these are much clearer than those detected in the spectral acquisition of the infrared data. Thus, Raman data used as input to generate the results reported in Figure 2 could perhaps present spectral artifacts due to thermal damage as each Raman and infrared spectrum obtained in this study represent the average spectrum from 10 spectra obtained in each scan. However, these changes are very subtle compared to the spectral changes associated with spectral shifts induced by the incorporation of heavy atoms, which end up dominating the information explained by PCA. Thus, spectral artifacts induced by thermal damage are not particularly relevant in the present study. However, caution must be taken when analyzing spectral data acquired via O-PTIR technology as spectral artefacts due to thermal damage can easily lead to data misinterpretation or misclassifications, especially when multivariate statistical methods are used to analyze samples with subtle biochemical differences as such techniques are able to identify small variations in the spectral signatures used as input. 

## 4. Conclusions

In conclusion, we have shown that a novel optical photothermal infrared spectrometer can be used for generating simultaneous infrared and Raman spectra from the same location from bacteria and that we could correlate this to the incorporation of ^13^C- and ^15^N-labeled compounds using simple chemometrics based on principal components analysis. PCA highlighted that the dominant change in the spectra was due to carbon-based functional group vibrations followed by nitrogen-based moieties, and this is not surprising as bacteria contain a relatively higher carbon content in comparison to nitrogen (C/N ratio 5.9 ± 1.1) [22]. As the *E. coli* cells are very small, with typical dimensions of 1 × 2 µm, and each contains just 1 pg biomass, care must be taken when multiple measurements are made from the same location. Although very minor in nature in this study, when PCA was used on a time series of continuous measurements from the same location, spectral artifacts could be seen as trends in PCA space and these are likely to be due to thermal damage, or at the very least from the sample increasing in temperature which may result in subtle effects on vibrational frequencies.

## Data Availability

The data that support the findings of this study are available from the first author (CL) on request.
